# Thermal Modeling of the Port on a Refining Furnace to Prevent Copper Infiltration and Slag Accretion

**DOI:** 10.3390/ma14226978

**Published:** 2021-11-18

**Authors:** Francisco José Jiménez-Espadafor Aguilar, José Antonio Vélez Godiño, Miguel Torres García, José María. Gallardo Fuentes, Eduardo Díaz Gutiérrez

**Affiliations:** 1 Departamento de Ingeniería Energética, Escuela Técnica Superior de Ingeniería de Sevilla, Universidad de Sevilla, Camino de los Descubrimientos, s/n, 41092 Seville, Spain; migueltorres@us.es; 2 Departamento de Máquinas y Motores Térmicos, Escuela Superior de Ingeniería, Universidad de Cádiz, Avda. Universidad de Cádiz, nº 10, Puerto Real, 11519 Cádiz, Spain; joseantonio.velez@uca.es; 3 Departamento de Ingeniería y Ciencia de los Materiales y del Transporte, Escuela Técnica Superior de Ingeniería de Sevilla, Universidad de Sevilla, Camino de los Descubrimientos, s/n, 41092 Seville, Spain; josemar@us.es (J.M.G.F.); eduardodiaz@us.es (E.D.G.)

**Keywords:** refining furnace, copper infiltration, thermal modeling, high-temperature heat transfer, model fitting

## Abstract

Fire refining of blister copper is a singular process at very high temperatures (~1400 K), which means the furnace is exposed to heavy thermal loads. The charge is directly heated by an internal burner. The impurities in the charge oxidize with the flux of hot gases, creating a slag layer on the top of the molten bath. This slag is periodically removed, which implies liquid metal flowing through the furnace port. To address its malfunction, a re-design of the furnace port is presented in this work. Due to the lack of previous technical information, the convective heat transfer coefficient between the slag and the furnace port was characterized through a combination of an experimental test and a three-dimensional transient model. Finally, the original design of the furnace port was analyzed and modifications were proposed, resulting in a reduction of the average temperature of the critical areas up to 300 K. This improvement prevents the anchoring of the accretion layer over the port plates and the steel plate from being attacked by the copper.

## 1. Introduction

A typical pyrometallurgical copper smelting process is developed in electrical or fire furnaces and includes three steps: smelting, converting, and fire refining. After the converting stage, the raw ore material is purified and produces blister copper that contains 98.5 to 99.5 percent copper. However, fire refining is needed in order to reduce the sulfur and oxygen content of the blister copper produced in Peirce–Smith converters. These processes require the addition of oxidizing agents and calcium and are developed at temperatures over 1373 K. In the case of fire furnaces, this high temperature is achieved through a combustion process between oxygen and natural gas as the fuel [1]. Fire refining is conducted in a furnace that is constructed with refractory-lined cylindrical steel shells mounted on trunnions at both ends, rotating around its longer axis for charging and pouring. An opening in the center of the furnace functions as a port through which blister copper, siliceous flux and scrap copper are charged [2]. The impurities are formed mainly by oxidized compounds and silicates that collect in a slag that floats over the purified melted copper [3,4]. In the furnace analyzed here, the port is opened around four times per day for approximately 10 min.

**Figure 1:**
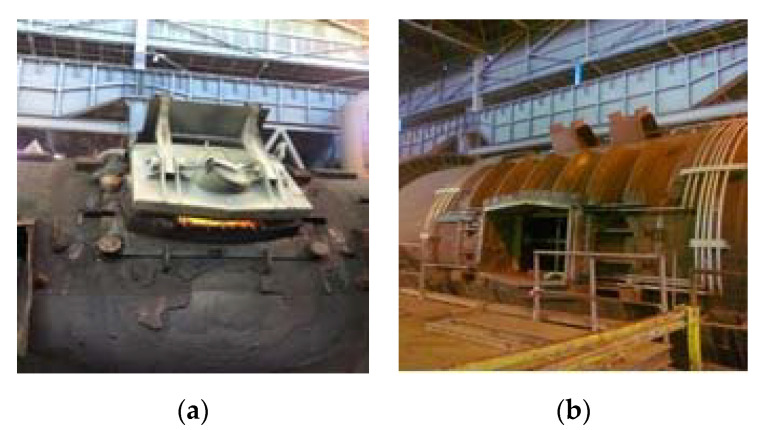
Layout of the furnace port. (**a**) Furnance in service; (**b**) maintenance shutdown.

Figure 1 presents the original layout of the furnace and the port that is integrated by six plates cooled by water. Figure 1a shows the furnace in service and the original port in the closed position, where improper sealing can be easily appreciated. This implies heat losses and the contamination of the working area with combustion gases. The lack of sealing is due to an accretion layer that adheres to the bottom plates of the furnace port. Figure 1b shows the furnace during a maintenance shutdown without the cover.

The plates of the port stand two different thermal loads. When the port cover is closed, the plates receive radiative flux from the burner flame, the melted copper and the remaining walls of the furnace, but with very low both surface area and view factor [5]. When the cover is opened, e.g., during the charging and pouring processes, a layer of slag or copper core at 1400 K flows over the plates. As a consequence, the pouring process imposes the highest thermal load to the bottom plates of the furnace port. Furthermore, a lack of information regarding the heat transfer from the slag flow to the plate was found, since no reference to the thermal characterization of this process was found in the technical literature. Hence, an experiment was designed to estimate the heat transfer coefficient [6].

**Figure 2:**
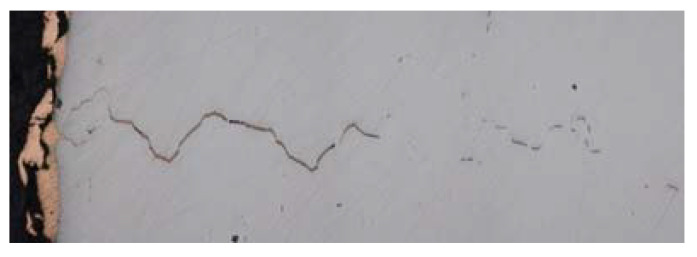
Copper infiltration into a S345J steel matrix.

The re-design of the filling/emptying port requires the following specifications:

- The external dimensions of the furnace port should remain unchanged.
- The port cover must be airtight to minimize hot gas leakages, preventing the contamination of the process area and heat losses during the pouring process [7]. Furthermore, these leaks boost the heat losses, which negatively impacts the fuel consumption. The energy conservation in this type of units represents one of the key topics regarding sustainable manufacturing processes [8,9,10].
- The new design shall guarantee a metal skin temperature below 750 K, to prevent copper infiltration into the steel plates [11]. Infiltration damages the base metal and may cause a catastrophic failure [12]. Figure 2 shows a micrograph of a S345J steel plate where the infiltration of copper can be appreciated. Figure 2 shows an approximately 2 mm infiltration, derived from a speed of 3 µm/s [13]. Although it has nearly no effect on properties such as the density or the specific heat, the infiltration process also increases the local conductivity of the metal, boosting the heat transfer [14].
- The proposed design must prevent the formation of an accretion layer of slag on the outlet area, which would impact the tightness of the port. This requirement is accomplished when the temperature of the bottom plates of the port is low enough so that any residue is dragged away during the pouring process.
- From the mechanical point of view, the port design must resist the thermal stress.
- The maintenance operations of the furnace port should be minimized to increase furnace availability.

This work characterizes the re-design of a furnace port, covering the experimental procedure developed to estimate the heat transfer coefficient between the slag and the port plates, the thermal analysis of the original port design and the discussion of the modifications constituting the new port design.

## 2. Materials and Methods

### 2.1. Original Design Characterization

**Figure 3:**
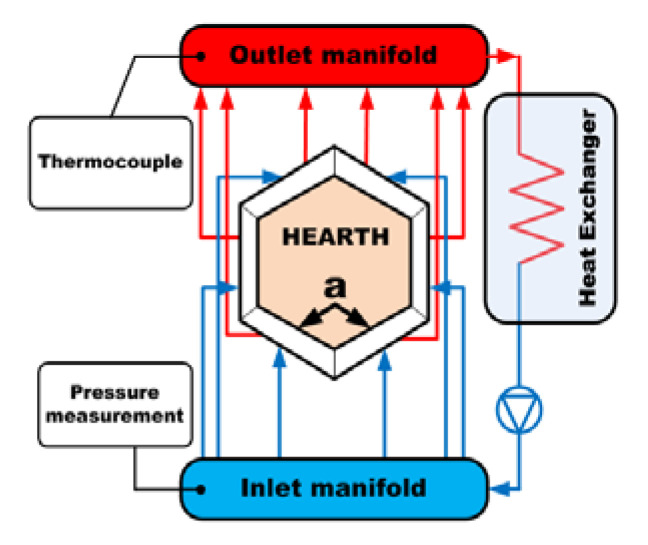
Scheme of the port cooling system.

One of the most restrictive specifications of the furnace port was the necessity of preventing the formation of an accretion layer of slag over the surface of the bottom plates. For this reason, reducing the temperature of the port plate surface as much as possible was required. Figure 3 shows the scheme of the original port cooling system. The six plates that form the furnace port had internal orifices that allow cooling water circulation, evacuating the heat transferred by the slag during the pouring process. Plates referred to as “a” in Figure 3 are particularly relevant, since they are in close contact with 1400 K slag during this operation. After leaving the port plates, the water flow (0.7 kg/s) was cooled in a cooling tower, reaching an almost constant temperature of 300 K downstream of the cooling water pump. The original cooling system had a temperature gauge in the outlet header, where the flow coming from the six plates mixed. Since each plate received a different heat flow, the bottom plates being higher, the temperature of the outlet header did not allow the evaluation of the temperature increase in the cooling water corresponding to each plate. Therefore, this average temperature underestimated the temperature of the cooling water coming from the bottom plates.

**Figure 4:**
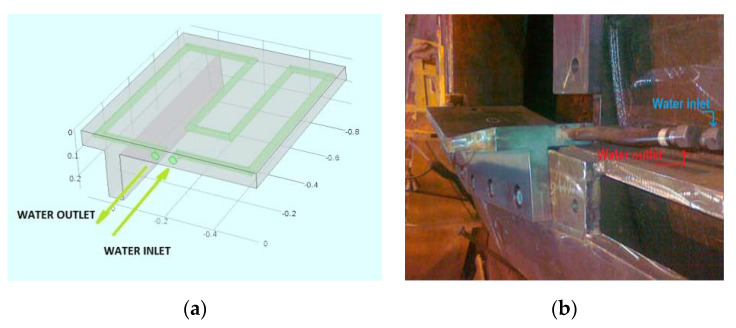
(**a**) Original plate design; (**b**) detail of the cooling water connections in the bottom plates.

Figure 4a shows a modeled view of the original design of the bottom plate, including the internal canalization for the cooling water. Furthermore, Figure 4b presents the two bottom plates installed on the furnace, showing a detail of the cooling water connections.

Despite the previously characterized features, the original furnace design failed in preventing the formation of an accretion layer on the bottom plate surface, dramatically increasing the maintenance cost of the furnace. Moreover, the increase in the cooling water flow up to the maximum allowable (1.4 kg/s) did not contribute to reduce the formation of the accretion layer. Consequently, a modification of the original design was required.

**Figure 5:**
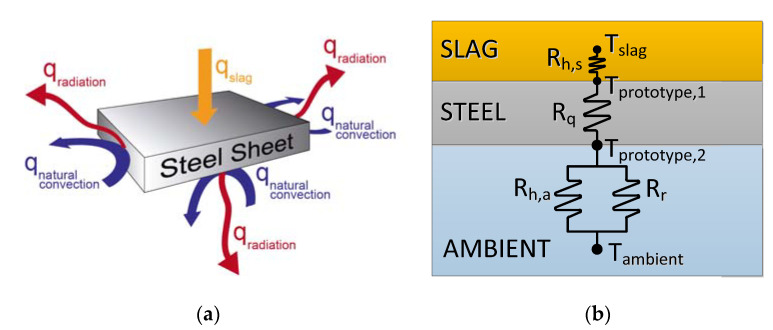
(**a**) Heat transfer mechanisms on port plates; (**b**) electrical analogy circuit.

To develop an alternative design of the port design, a finite element model was developed in ANSYS CFD software (version 2020). Figure 5a presents the different heat transfer mechanisms that occur for the analyzed plates of the furnace port: radiation and convective heat flux between the plates and the surroundings [15], conduction through the metal and, finally, convective heat transfer from the slag. Additionally, Figure 5b shows the equivalent resistance circuit derived from the electrical analogy to clarify the heat transmission process.

The previously referred finite element model requires the data corresponding to the convective heat transfer coefficient, as derived from Figure 5b. However, no applicable reference was found in the technical literature, so an ad hoc procedure was necessary to carry out its proper estimation. Hence, the operational conditions of the furnace port were experimentally reproduced, specifically when the port is opened and the slag is poured outside the furnace. This allowed the surface actual temperature of the bottom plates of the port to be characterized. Subsequently a numerical model was used to match the trace of this experimental surface temperature, allowing the identification of the representative convective heat transfer coefficient.

### 2.2. Experimental Prototype

**Figure 6:**
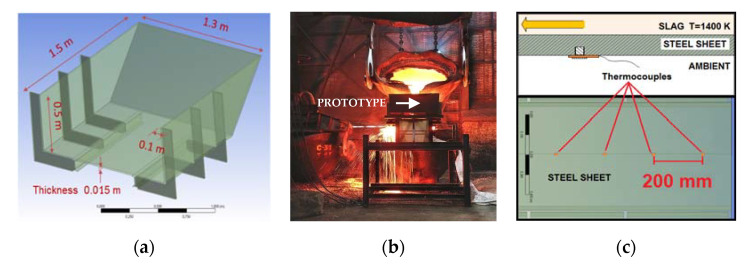
Layout of the prototype and the experiment. (**a**) Prototype main dimensions; (**b**) prototype during the test; (**c**) location of the thermocouples.

To characterize the actual temperature of the bottom plates of the port, a prototype based on a 15 mm thickness S275JR steel plate was built. The prototype main dimensions are characterized in Figure 6a. The slag at 1400 K was poured over the prototype from a 10 m^3^ crucible. During the experiment, the slag flowed and completely covered the bottom plate of the prototype, creating a layer equivalent to the actual layer created during furnace pouring (35–50 mm thick and 0.5–0.6 m/s). Figure 6b shows a picture of the prototype during the test, where the pouring of the slag from the crucible to the prototype can be observed. Finally, Figure 6c presents side and plant views of the prototype, including the location of the four evenly spaced thermocouples to measure the time-dependent temperature evolution on the back of the prototype. Type N thermocouples were used and the data logger acquired data at 1 kHz frequency.

The experiment started with the entire prototype at room temperature (300 K). Due to the limited volume of the poured slag, the speed of the pouring process and the very large thermal gradient between the slag and the prototype at the beginning, the thermal answer of the prototype did not reach stationary conditions during the experiment. Regarding the temperature evolution of the four thermocouples installed in the prototype (refer to Figure 6c), two trends were identified: first a rapid increase in the temperature, followed by an abrupt change in the slope when the available slag was consumed.

### 2.3. Numerical Model

A transient finite element model was developed to evaluate the convective heat transfer coefficient between the slag and the port metal [16,17]. The overall heat transfer process is controlled by the heat transfer from the slag and the radiative heat loss to the surroundings, the convective heat transfer to the air being less relevant [18]. As stated in Figure 5b, the heat flux between the ambient and the prototype lower surface (q, Equation (1)), combines convective ($q_{conv}$, Equation (2)) and radiative ($q_{rad}$, Equation (3)) transfer mechanisms.

(1) $$q=q_{conv}+q_{rad}$$

(2) $$q_{conv}=h_{amb}\cdot \left(T_{p,2}-T_{amb}\right)$$

(3) $$q_{rad}=\varepsilon \cdot \sigma \cdot \left(T_{p,2}^{4}-T_{amb}^{4}\right)$$

Constants involved in the previous equations were the ambient temperature $T_{amb}=300\;K$, the convective heat transfer coefficient hamb=12Wm2K, the metal emissivity ε=0.9 and, finally, the Stefan-Boltzmann constant σ=5.67×10−8 Wm2K4. Additionally, the temperature of the prototype lower surface $T_{p,2}$ directly derived from the experimental procedure described in Section 2.2, allowing the characterization of the heat flux through the metal plate. The transient conduction through the plate was modeled by the Fourier equation (Equation (4)), where metal conductivity (K), density (ρ) and thermal capacity (Cp) correspond to S275JR steel:

(4) $$\frac{\delta T}{\delta t}=\frac{K}{\rho \cdot Cp}\cdot \nabla^{2}T$$

Finally, the modeling of the convective heat transfer from the slag to the metal ($q_{slag}$, Equation (5)) allowed the characterization of the convective coefficient ($h_{slag}$), since both the slag temperature ($T_{slag}$) and the temperature of the prototype upper surface ($T_{p,1}$) were known:

(5) $$q_{slag}=h_{slag}\cdot \left(T_{slag}-T_{p,1}\right)$$

The slag temperature was assumed to be constant ($T_{slag}=1400\;K$) during the experiment due to its high thermal capacity and the low resident time over the plate (<2 s).

After modeling the heat transfer mechanisms, the convective heat transfer coefficient between the slag and the metal ($h_{slag}$) can be characterized. For this purpose, an iterative procedure was applied to identify the minimum of the function error (Equation (6)):

(6) $$Error=\sqrt{\overset{t_{end}}{\underset{t_{in}}{\sum }}{\left[T_{p,2}\left(h_{slag},t\right)-\frac{1}{4}\cdot \overset{4}{\underset{n=1}{\sum }}T_{gauge,n}\left(t\right)\;\right]}^{2}}$$

**Figure 7:**
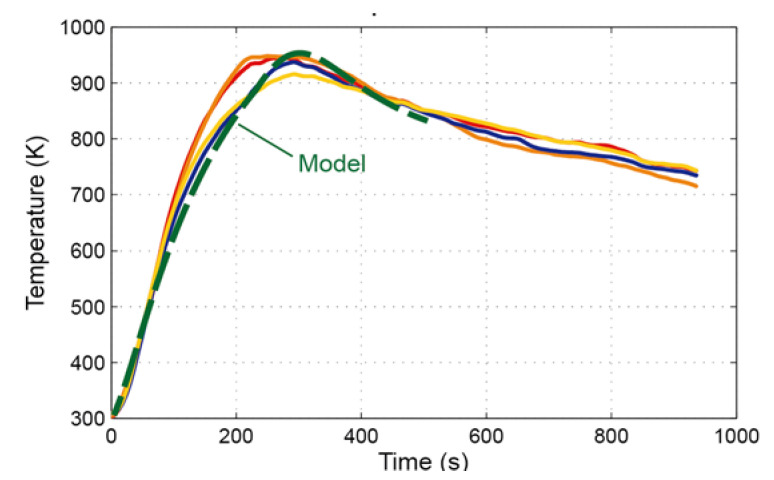
Temporal evolution of the temperatures in the prototype, including the model output (hslag=260Wm2K).

Equation (6) evaluates the deviation between the temperature of the lower surface provided by the model (calculated in an intermediate point among the four thermocouples) and the average temperature from the four thermocouples installed in the prototype. To conclude with the calculation of the convective coefficient, Figure 7 shows the temporal evolution of the temperatures measured in the four thermocouples installed in the prototype, including the model output for the optimal value of $h_{slag}$ (260Wm2K). A sensitivity analysis was performed to analyze the dependence of the optimal $h_{slag}$ within the typical range of $h_{amb}$ [19], resulting in a negligible dispersion of the optimal $h_{slag}$. Finally, the slight mismatch observed in the temperature evolution between the experiment and the model results was irrelevant for the evaluation of alternative designs of the furnace port. Moreover, although no reference regarding the convective heat transfer coefficient between the copper slag and the furnace plates was found, values ranging from 120 to 450 W/m^2^K are estimated for molten steel [20,21].

Once the previous experimental procedure allowed the characterization of a representative convective heat transfer coefficient between the slag and the metal ($h_{slag}$), the evaluation of alternative designs of the furnace port was addressed. Based on the actual slag pouring process duration, the three-dimensional model used for this purpose was stationary. Moreover, the differences regarding the previous transient model were the following:

- Differently from the previous procedure, this analysis considered a constant $h_{slag}$ to calculate the metal temperatures, especially the temperature of the plate upper surface ($T_{p,1}$). The slag temperature was not modified ($T_{slag}=1400\;K$).
- For safety reasons, the convective heat transfer coefficient between the slag and the metal was increased by 50 percent, considering the uncertainties in its estimation.
- The stationary conduction through the plate was modeled by the Laplace equation (Equation (7)), with imposed heat flux (Fourier law, Equation (8)):

(7) $$\nabla^{2}T=0$$

(8) $$q_{cond}=-K\cdot \nabla T$$

- Port lateral surfaces and other surfaces in contact with furnace inner parts were considered thermally insulated.
- A Reynolds number (Re) > 30,000 was within the expected ranges of water mass flow, water temperature and internal channels characteristic length. Therefore, the flow was fully turbulent and far from laminar conditions. The modeling was carried out using the standard Fluent code for fluid heat transfer problems through the K-ε turbulence model [22,23,24]. Different grid densities were evaluated, but the low fluid speed and the large thermal gradient resulted in a very low sensitivity of the temperature field to the grid density, as observed for similar heat transfer analysis [25].

## 3. Results and Discussion

**Figure 8:**
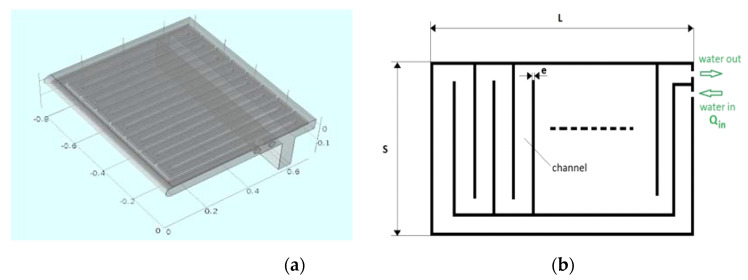
(**a**) A 3D model for the new plate design; (**b**) sketch of the cooling channels.

Figure 8 shows the new concept for the port plate, where the overall external dimensions were not changed, but the heat transfer area was increased approximately 60 percent. In the original design, the internal channels were drilled in a massive block. However, in the proposed concept, the plate was built by welding steel plates that formed a continuous serpentine through which the water was circulated. This channel practically covered the complete internal surface of the port plate, significantly increasing the global heat transfer due to the following factors:

- An increase in the heat transfer area between the upper plate and the cooling water.
- A reduction in the conduction thermal resistance due to the decrease in the plate thickness.

Three parameters were considered as independent variables for the new port design: the plate thickness (e), the number of serpentine channels (N) and, finally, the cooling water mass flow ($Q_{in}$). Additionally, the external dimensions of port plates were not allowed to be modified.

**Figure 9:**
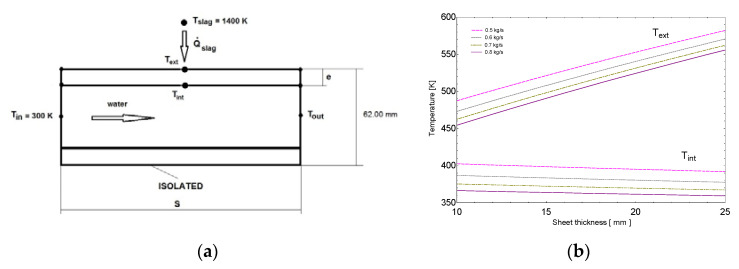
(**a**) Simplified heat transfer model; (**b**) temperature output.

To establish a preliminary estimation of the influence of the mentioned design parameters over the upper surface temperature of the port plate, a simplified two-dimensional model was developed (Figure 9). This model was integrated by a semi-infinite plate of dimension S, with constant both plate thickness and channel area and variable water mass flow and internal sheet thickness (e). As in previous models, the heat was transferred from the slag at a constant temperature of 1400 K with hslag=260Wm2K. Regarding the convective heat transfer, the cooling water flow was considered fully developed in the turbulent regime [26]. In Figure 9b, where the cooling water inlet temperature is 300 K, parameters $T_{ext}$ and $T_{int}$ are the average values of metal temperature in the upper and lower side of the plate, respectively. The transport properties of water were evaluated at the water average temperature (Tin+Tout2), being nearly constant over the analyzed range. Finally, the Reynolds number was far greater than 30,000 for the tested conditions. The preliminary conclusions derived from the results of the simplified two-dimensional analysis (Figure 9b) were the following:

- Regardless of the cooling water mass flow, an increase in the sheet thickness implied an increase in $T_{ext}$ and a reduction of $T_{int}$, therefore diminishing the thermal energy extracted from the plate.
- An increase in the mass flow of cooling water slightly reduces both $T_{ext}$ and $T_{int}$.
- Finally, the higher the sheet thickness, the less effective the increase in the mass flow of cooling water.

Because the main goal of the new design was to reduce $T_{ext}$ to prevent anchoring of the accretion layer, the results of the simplified analysis allowed concluding that the optimal solution combines the minimum feasible sheet thickness and the maximum cooling water mass flow. However, the higher the mass flow, the higher the power consumed by the hydraulic system, not only due to the increase in the mass flow, but also to the increase in the pressure losses. Therefore, the power demanded by the proposed design must be within the available capacity of the original hydraulic system.

**Figure 10:**
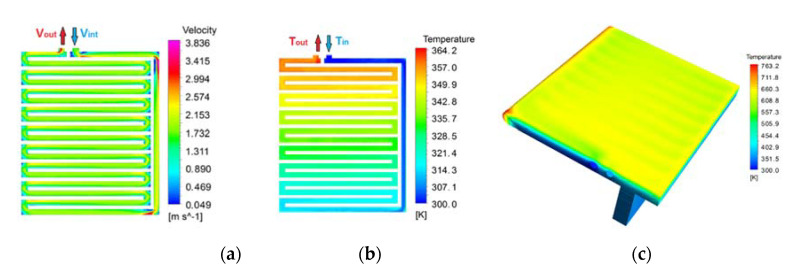
(**a**) Detailed model results: water speed; (**b**) cooling water temperature; (**c**) metal temperature.

Figure 10 shows the outputs of the stationary 3D model for the proposed alternative design, based in 17 channels (N), sheet thickness of 20 mm (e) and cooling water mass flow of 0.7 kg/s ($Q_{in}$). As expected, the cooling water speed (Figure 10a) was almost constant except in the corners, where the deviations regarding the average speed were identified. The cooling water temperature (Figure 10b) increased in almost a linear trend from the inlet to the outlet. The external metal temperature (Figure 10c) was almost uniform (~630 K), although the effect of the channels can be appreciated due to a small thermal difference. As expected, the highest temperatures were located at the internal edges, where the surface-to-volume ratio was higher. For this reason, these areas were rounded, as shown in Figure 8a.

**Figure 11:**
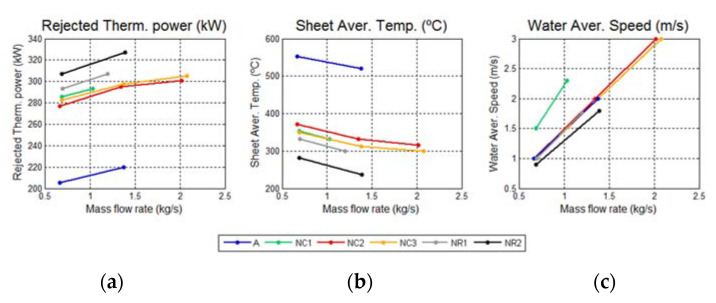
(**a**) Port design comparison: cooling thermal power; (**b**) upper plate average temperature; (**c**) cooling water average speed.

The main results for the original design (A) and for different proposed configurations (rounded NC and non-rounded NR) are shown in Table 1 and Figure 11.

The advantages of the proposed designs over the original approach are clearly shown in Figure 11a,b, since the new designs increased the cooling thermal power between 35 and 50 percent. This implied a reduction in the metal external temperature in the alternative designs. Moreover, the new designs reduced the maximum metal temperature ($T_{metal}^{max}$) by more than 300 K compared to the original design, which minimized the potentiality for copper infiltration.

The parameter $T_{metal}^{avg}$ provides the average temperature on the upper surface of the metal plate and, therefore, is the most representative parameter for the evaluation of the potential of the proposed designs to prevent the accretion layer anchoring. According to these results, the lower the sheet thickness, the lower $T_{metal}^{avg}$, regardless of the cooling water mass flow. In particular, for the case of e = 10 mm, the reduction in $T_{metal}^{avg}$ regarding the original solution was approximately 300 K. Additionally, the higher the cooling water flow rate, the lower $T_{metal}^{avg}$, due to the increase in the convective heat transfer coefficient. However, this trend was asymptotic for flows higher than 2.5 kg/s, since the controlling thermal resistance became that associated to the conductive heat transfer.

Regarding $T_{water}^{out}$, as preliminarily expected, the lower the water flow rate, the higher the water outlet temperature for all the analyzed designs. If the absolute water pressure decreased below the saturation pressure evaluated at $T_{water}^{out}$, water bubbles formed within the flow, drastically decreasing the heat transfer coefficient and, therefore, increasing the local temperature of the metal [27]. This situation may produce a catastrophic failure. Furthermore, in the serpentine there were zones with a high water speed and, therefore, with a relatively low local pressure. Analyzing Figure 10a,b some areas with high water speed (low pressure) and high water temperature were identified, constituting the worse combination from the cavitation point of view and resulting in high metal temperatures. An increase in the metal temperature had two undesirable effects: first, a decrease in the steel mechanical properties (Young’s modulus and yield limit) and, second, the increase in the copper infiltration into the steel matrix, both increasing the risk of a failure in the design. However, the cavitation in the serpentine was prevented if the cooling water circuit was pressurized over 3 bar.

## 4. Conclusions

A valid methodology, based on the combination of a 3D transient model and a dedicated experimental test rig, was established for a characterization of the convective heat transfer coefficient between the flowing slag and the metal plates comprising the furnace port, applicable to similar systems. Specifically, this approach resulted in hslag=260Wm2K, with a very low sensitivity to the natural convection coefficient between the metal and the ambient air.

Both, an increase in the transfer area between the cooling water and the metal plates and a reduction in the plates thickness was enough to improve the original design for the furnace port. This alternative design reduced the average metal surface temperature by 300 K regarding the original design, while the maximum temperature decreased to 750 K, which consequently prevented the risk of copper infiltration into the steel plates. Finally, the lower temperatures achieved at the upper port plates prevented anchoring of the accretions, decreasing the maintenance costs of the furnace.

To conclude, the positive contribution of the alternative designs to the sustainability of the refining process is noteworthy. This idea is mainly supported by the potential elimination of hot gas leakages through the furnace port, which are a source of both uncontrolled polluting emissions (mainly CO_2_ and NOx) and heat losses. Consequently, the proposed designs not only improve mechanical integrity of the furnace but also the consumption of primary energy is reduced.

## Figures and Tables

**Table 1:** Port design comparison.

Design	Sheet Thickness (mm)		N	Water Flow (kg/s)	$T_{metal}^{max}$ (K)	$T_{metal}^{avg}$ (K)	$T_{water}^{out}$ (K)	$∆T_{water}$ (K)	Avg. Water Speed (m/s)
	Inn. Channel	Ext. Channel							
Original	21	-	-	0.67	1131.2	825.2	373.5	346.6	1.0
Original	21	-	-	1.38	1115.2	794.2	338.2	311.3	2.0
Round corners	20	10	25	0.69	784.2	626.8	399.1	372.3	1.5
Round corners	20	10	25	1.03	766.2	605.6	368.2	341.3	2.3
Round corners	20	20	17	0.67	810.2	643.9	399.1	372.3	1.0
Round corners	20	20	17	1.35	772.2	605.9	352.2	325.5	2.0
Round corners	20	20	17	2.02	754.2	589.4	335.6	308.8	3.0
Round corners	18	18	19	0.69	772.2	623.7	398.2	371.2	1.0
Round corners	18	18	19	1.38	736.2	585.4	351.7	324.8	2.0
Round corners	18	18	19	2.08	720.2	573.3	335.2	308.3	3.0
Straight corners	15	15	23	0.70	821.2	604.2	399.7	373.2	1.0
Straight corners	15	15	23	1.20	794.2	572.0	361.2	334.4	1.8
Straight corners	10	10	29	0.69	637.2	555.2	406.5	379.7	0.9
Straight corners	10	10	29	1.39	587.2	510.2	356.4	329.5	1.8
